# Subcapsular Kidney Urinoma After Percutaneous Nephrolithotomy

**DOI:** 10.1089/cren.2017.0011

**Published:** 2017-04-01

**Authors:** Eugenio Di Grazia, Letterio D'Arrigo, Alberto Saita, Giuseppe Giordano, Giuseppe Russo, Giuseppe Morgia, Pasquale La Rosa

**Affiliations:** ^1^ Urology Unit, Garibaldi Hospital, Catania, Italy.; ^2^ Urology Unit, Cannizzaro Hospital, Catania, Italy.; ^3^ Department of Urology, Humanitas Clinical and Research Center, Humanitas University, Rozzano, Italy.; ^4^ Radiology Unit, Garibaldi Hospital, Catania, Italy.; ^5^ Department of Urology, University of Catania, Catania, Italy.

**Keywords:** percutaneous nephrolitothomy, PCNL complication, kidney trauma, urinoma

## Abstract

***Background:*** A rare percutaneous nephrolithotomy (PCNL) complication and its management is reported.

***Case Presentation:*** A male patient, 43 years of age, underwent PCNL for a large left pyelocaliceal stone. Surgery was performed in the Valdivia–Galdakao supine position. The percutaneous tract was established by combined radiologic and sonographic guidance. The tract was dilated by balloon and a 24F Amplatz sheath was located. As complete clearance was not achieved because of a residual lower pole caliceal stone, a ureteral Double-J and a 20F nephrostomy were located for a second-look PCNL through the same tract after 7 days. After second-look PCNL residual stone was still not cleared because it was unreachable through the tract established and the patient was discharged without nephrostomy and with the ureteral stent, retrograde intrarenal surgery (RIRS) was planned in 3 to 4 weeks. Hemoglobin, hematocrit, and the renal function were normal. On the seventh day after PCNL, no leakage was detected from the percutaneous tract, but the patient started to complain about flank discomfort and fever. Imaging showed a 6 cm lower pole subcapsular collection. After 3 days of conservative management with antibiotics, the subcapsular collection did not resolve and a percutaneous 6F mono-J drainage in the collection was placed. Drain output was at first purulent and evolved into urine throughout the following days. Drain urine culture was positive for *Escherichia coli* infection and carbapenemic-targeted antibiotic was offered to the patient. Collection drained about 400 cc in 7 days and the drain was removed when the output was less than 10 cc per day. No late complications were reported and RIRS was scheduled in 1 month to clear the residual stone.

***Conclusion:*** Subcapsular urinoma post-PCNL is an uncommon but severe complication. Prompt and correct drainage may solve it.

## Subcapsular Kidney Urinoma After PCNL

Percutaneous nephrolithotomy (PCNL), as primary treatment of kidney urinary stones, has regained much interest in the past decade, thanks to the variations and refinements of the technique. Although 54% of complications are negligible, such as fever and small bleeding, for which no invasive intervention is needed (I grade according to the Clavien-Dindo classification), severe complications may occur and a prompt and correct management should be established to avoid the worsening of patient clinical state.^1^ We report on an unusual PCNL complication and its management.

## Case Presentation

### Clinical history

A 43-year-old man came to our Institution to undergo a PCNL procedure. A ureteral Double-J stent had already been placed in another Institution 6 months before to relieve symptomatic hydronephrosis. His previous surgery included three left ureteroscopies and four left renal extracorporeal lithotripsies for urinary stones. No other relevant pathologies emerged from his medical history and no previous metabolic analysis was carried out.

### Physical examination

The patient was 182 cm tall and his body mass index was 22.6 kg/m^2^. Vital signs were normal. Routine preoperative work-up and urine culture were normal.

### Diagnosis

**FIG. 1.**
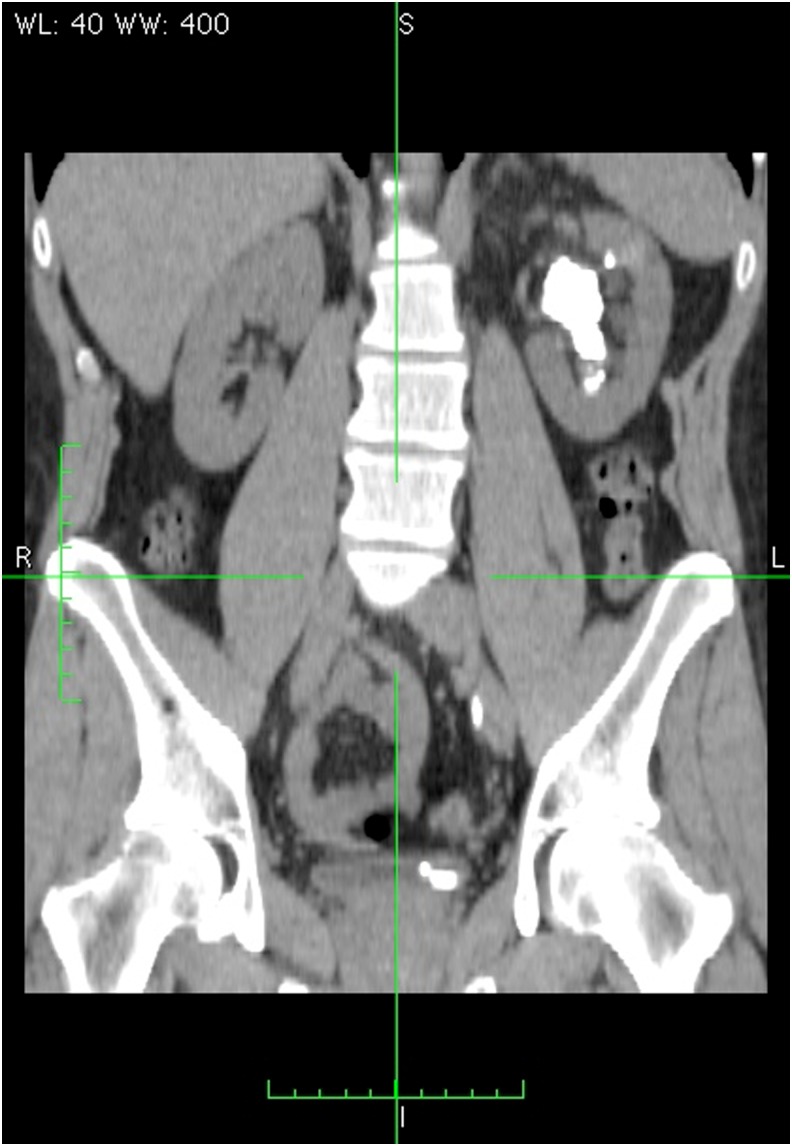
Coronal CT scan showing pretreatment staghorn left kidney stone.

Preoperative noncontrast CT scan revealed multiple and large stones, involving left renal pelvis and lower pole calix (Fig. 1). Pelvic stone was about 4 cm in length whereas caliceal stone was 1.5 cm in length. Mild hydronephrosis was present and density of stones was about 1100 Hounsfield units.

### Intervention

The patient was placed in the Galdakao-modified supine Valdivia position.^2^ After Double-J stent removal, retrograde pyelography was carried out after placement of an open-ended ureteral catheter 5F (Boston Scientific^®^) under fluoroscopic guidance. A percutaneous renal access was carried out by puncturing a medial-posterior kidney calix with a Chiba needle 18-gauge under biplanar fluoroscopic and ultrasound guidance. The tract was dilated to 24F by using a balloon (BARD X-Force^®^) and an Amplatz working sheath 24F was located. The stone was fragmented using combined ballistic and ultrasound (SWISS LITHOCLAST**-**MASTER^®^). The stone fragments were extracted by using a 10F extractor (Perc N-Circle**-**COOK^®^). A 1.5 cm lower pole stone could not be removed by the ante grade approach because it was located in a calix not aligned to the access accomplished. Although the Validivia–Galdakao position usually allows for combining retrograde and antegrade approaches and at the same time improving stone-free rate, retrograde management of the residual stone during the same session was not attempted because of poor ureteroscopic vision for little bleeding. At the end of the procedure, a Double-J ureteral stent 6F (Boston Scientific) and a nephrostomy tube 20F (Soft Drain BARD^®^) were placed for a second-look PCNL. The time of the operation was about 90 minutes and blood loss was negligible during the surgery. Laboratory studies showed stable levels of hemoglobin, hematocrit, and renal function.

### Follow-up

The following postoperative 7 days were uneventful and the patient was scheduled for the residual stone treatment through the same path accomplished before. In our practice, quick PCNL second looks are usually performed under sedation without an Amplatz sheath and by using a 15 flexible nephroscope, Holmium laser to fracture stones, and no-tip baskets to retrieve fragments. The procedure is usually completely nontraumatic because the discrepancy between the 20F mature tract and the 15F flexible nephroscope allows, immediately after the tube removal, to get through the path to the kidney collecting system without any friction and patient discomfort. One centimeter length residual stone was still present in a lower calix because it was unreachable through the path established and the patient was discharged with the only Double-J stent the day after. The plan was to perform a retrograde intrarenal surgery (RIRS) in 3 to 4 weeks to complete the treatment.

### Outcomes

**FIG. 2.**
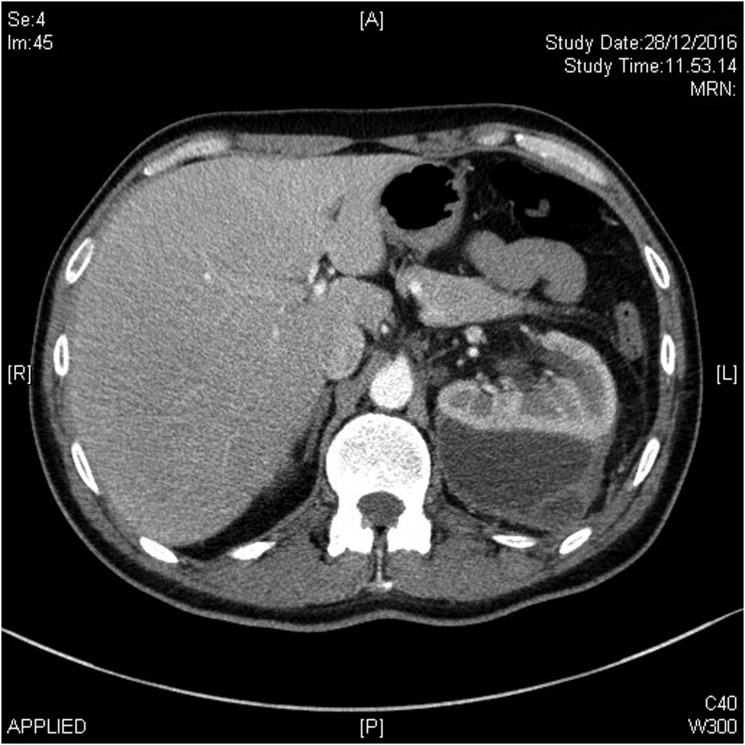
CT scan showing subcapsular kidney collection after second-look percutaneous nephrolithotomy. No external or retroperitoneal extravasation is observed.

**FIG. 3.**
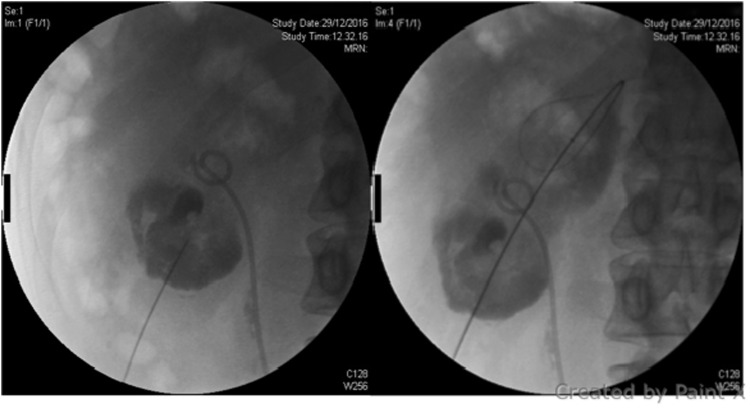
Subcapsular kidney collection puncture and mono-J drain placement under sonographic and radiologic guidance as performed by the interventional radiologist.

**FIG. 4.**
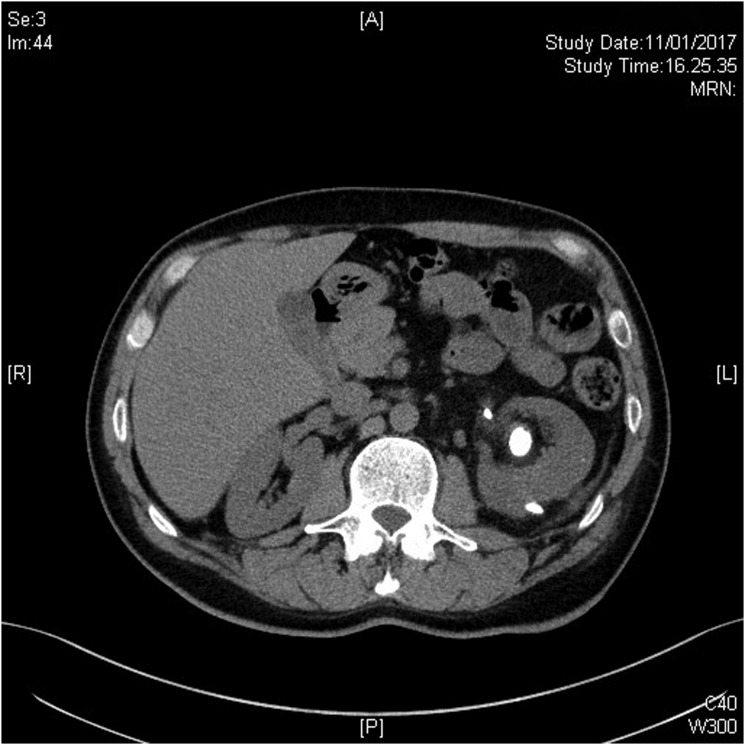
CT scan showing subcapsular kidney collection healing after 7 days of drainage.

On the 7th day after surgery, the tract was completely sealed but the patient started to complain about flank discomfort and fever. Hemoglobin, hematocrit, and renal function were stable. Ultrasonography and CT scan with intravenous contrast showed a 6 cm subcapsular intraparenchymal collection. A belated sequence of CT scan showed accumulation of contrast into the collection. After 3 days of conservative management with ciprofloxacin 1 g per day, the subcapsular collection did not resolve (Fig. 2). The patient was readmitted and a percutaneous 6F mono-J drainage was located in the collection under a combined ultrasonographic and radiologic guidance by an interventional radiologist (Fig. 3). Although the first output from the drainage was purulent, later on it turned into clear urine throughout the days after. No bloody output was observed neither when the drain was placed nor in the days after, and the hemoglobin and hematocrit remained stable. Culture analysis was positive for *Escherichia coli* infection and carbapenemic-targeted antibiotic was offered to the patient until drainage removal. The patient was discharged after 2 days when the fever and the pain ceased. From the collection, about 400 cc was drained in 7 days and the drainage was removed after 8 days when the liquid output was <10 cc per day (Fig. 4). The patient recovered and RIRS was scheduled in 1 month to manage the residual stone.

## Discussion and Literature Review

Improvement of surgical care demands transparent, consistent, and accurate reporting of surgical outcomes that are evaluated and documented in a standardized manner.

A Clavien–Dindo complication classification has recently been adopted and validated in a PCNL surgery. Categorization of PCNL-specific complications according to Clavien classification score based on expert opinions collected from 74 urologists through an international survey has mentioned most of the PCNL complications and relative management.^3^ In the Clavien–Dindo classification, it may be located at the 3B category, because its resolution needed a radiologic intervention under local anesthesia. To our knowledge and after a literature revision on this topic, the aforementioned complication and its pathogenic formation mechanism are not mentioned and deserve to be reported.^4^ Penetrating traumas can cause urinomas by two mechanisms: direct disruption of the pelvis or collecting system or by degeneration of nonviable tissue. These urinomas are often perinephric, but can also occur in a subcapsular location. The subcapsular collection did not resolve spontaneously because an internal fistula between the damaged fornix and the subcapsular space supplying the collection had been established as a consequence of incorrect calix puncture and dilatation. The second-look PCNL irrigation without an Amplatz sheath probably plumped the collection through the fistulous small path, although it was carried out 1 week later when the tract should have been mature enough and the damaged calix healed. The collection should usually resolve without further management when the collecting system is adequately drained by a stent, prompting for the tract sealing in few hours after nephrostomy removal. Percutaneous drainage of the urinoma itself is not always necessary because spontaneous reabsorption can occur if the leakage has stopped. Assuming the source of urinoma can heal spontaneously, conservative management may be all that is required. That is the reason why we started managing the complication conservatively. Nevertheless the percutaneous tract sealed quickly and the subcapsular collection continued being supplied by the internal fistula. After 7 days, the patient became symptomatic as the collection augmented and evolved into an abscess. Needle aspiration of the urinomas may suffice if the underlying problem is sufficiently addressed. However, a catheter can usually more completely drain the urinoma and allows monitoring of output to determine whether the underlying problem has been adequately treated. In addition, catheter placement maintains access into the collection until culture and fluid analysis has been completed. Drainage can be easily accomplished in most cases by ultrasound-guided placement of a drain. Because these are often retroperitoneal in location, there is usually a clear window into the subcapsular urinoma. Output from the drain should be monitored and the catheter removed when the output is consistently <10 cc per day. If the underlying source of urine leakage is adequately controlled, complete drainage should be accomplished within a few days. Another interesting aspect was the complete absence of blood clots in the collection such as when scapular hematoma is concerned, but rather a urine extravasation supplied by the second-look PCNL irrigation. Even in experienced hands, incorrect calix puncture together with dilatation may severely complicate PCNL.

## Conclusion

To our knowledge, subcapsular urinoma is a rare and serious complication after PCNL surgery. Prompt detection and minimally invasive management may solve this complication.

## Abbreviations Used

CT: computed tomography
PCNL: percutaneous nephrolithotomy
RIRS: retrograde intrarenal surgery

## References

[1] Labate G, Modi P, Timoney A, Cormio L, Zhang X, Louie M, Grabe M, de la Rosette J; on behalf of the CROES PCNL Study Group J. The percutaneous nephrolithotomy global study: Classification of complications. J Endourol 2011;25:1275–1280 21751882 10.1089/end.2011.0067

[2] Ibarluzea G, Scoffone CM, Cracco CM, et al. Supine Valdivia and modified lithotomy position for simultaneous anterograde and retrograde endourological access. BJU Int 2007;100:233–236 17552975 10.1111/j.1464-410X.2007.06960.x

[3] de la Rosette JJ, Opondo D, Daels FP, et al.; CROES PCNL Study Group. Categorisation of complications and validation of the Clavien score for percutaneous nephrolithotomy. Eur Urol 2012;62:246–255 22487016 10.1016/j.eururo.2012.03.055

[4] Labate G, Modi P, Timoney A, Cormio L, Zhang X, Louie M, Grabe M, Rosette; On Behalf Of The CROES PCNL Study Group J. The percutaneous nephrolithotomy global study: Classification of complications. J Endourol 2011;25:1275–1280 21751882 10.1089/end.2011.0067

